# Hepatic adenomatosis leading to liver failure and symptomatic extrinsic cardiac compression treated with liver transplantation: a case report and literature review

**DOI:** 10.3389/frtra.2026.1810971

**Published:** 2026-06-03

**Authors:** Antoine Robert, Nicole Bhave, Jared Christensen, Kyle Sheetz, Nicole Tomm, Robert John Fontana

**Affiliations:** 1 Toronto Center for Liver Disease, University of Toronto, Toronto, ON, Canada; 2 Division of Cardiology, University of Michigan, Ann Arbor, MI, United States; 3 Division of Interventional Radiology, University of Michigan, Ann Arbor, MI, United States; 4 Division of Transplant Surgery, University of Michigan, Ann Arbor, MI, United States; 5 Department of Pathology, University of Michigan, Ann Arbor, MI, United States; 6 Division of Gastroenterology and Hepatology, University of Michigan, Ann Arbor, MI, United States

**Keywords:** adenoma—pathology, liver adenoma, liver failure, liver transplant, radioembolization hépatique

## Abstract

We report an unusual case of a 34-year-old woman with liver and right heart failure caused by mass effect from a large hepatic adenoma compressing the bile duct, right atrium, and vena cava that was successfully managed with sequential radioembolization and orthotopic liver transplantation. This case highlights the potential complications of large adenomas and associated downsizing strategies needed to achieve a successful outcome. We believe that this is a topic of interest to the journal readership as hepatic adenomatosis is a rare indication for liver transplantation that many readers may not be familiar with.

## Introduction

Hepatic adenomatosis is an infrequent indication for liver transplantation (LT) with only 142 liver transplants in the United States reported to UNOS from 1994 to 2020 (1). Herein, we report an unusual case of a 34-year-old woman presenting with liver and right heart failure symptoms caused by mass effect from hepatic adenomatosis that was successfully managed with radioembolization and subsequent LT. To provide clinical context, we also provide a synopsis of other published case series.

## Case presentation

A 34-year-old white woman with obesity (a body mass index of 32 kg/m^2^) and systemic lupus erythematosus (SLE) was hospitalized for new-onset painless jaundice over the past month. She was initially diagnosed with multiple liver adenomas at age 18 on contrast-enhanced CT scans while receiving oral contraceptive pills. Three years after discontinuation of the oral contraceptives, she had a 12 cm adenoma of the right lobe and three additional adenomas with a normal serum alpha-fetoprotein (AFP). Enucleation of the segment 3 adenomas and right hemihepatectomy demonstrated 11.5, 5, and 3.8 cm benign adenomas without beta-catenin expression. Postoperatively, she underwent serial liver ultrasounds for 2 years and SLE was managed with hydroxychloroquine and belimumab injections. At age 34, she had a serum aspartate aminotransferase (AST) of 41 U/L, alanine aminotransferase (ALT) of 80 U/L, and a detectable antinuclear antibody and antimitochondrial antibody, and a liver ultrasound demonstrated increased echotexture but no focal masses. A liver biopsy to exclude autoimmune liver disease demonstrated grade 2–3 metabolic dysfunction–associated steatohepatitis (MASH) with periportal fibrosis, following which diet and exercise were recommended.

Six months later, she was hospitalized with new-onset jaundice and epigastric abdominal pain with a total bilirubin 12.3 mg/dL (direct at 9.2 mg/dL), alkaline phosphatase 237 U/L, AST 77 U/L, ALT 96 U/L, and an international normalized ratio (INR) of 1.2. A liver MRI (Figure 1) with contrast revealed multiple hepatic adenomas replacing 50% of the residual hepatic parenchyma, with the largest lesion measuring 19.2 cm, displacing the right hemidiaphragm upward. The right atrium and right ventricle were partially collapsed on MRI imaging by the protruding mass, which was confirmed by two-dimensional echocardiography. The lower tip of the mass in the caudate also caused partial obstruction of the inferior vena cava. The patient experienced anasarca as a consequence of extrinsic compression and impaired filling of the right heart, but there was no evidence of complete cardiovascular collapse, and she remained normotensive. Serum tumor markers such as AFP, CEA, and CA19-9 were all within normal limits and the patient was managed with analgesics and bed rest. An ERCP demonstrated displacement of the left main duct and rarefaction of intrahepatic ducts due to the right lobe mass, but there was no overt biliary obstruction.

**Figure 1:**
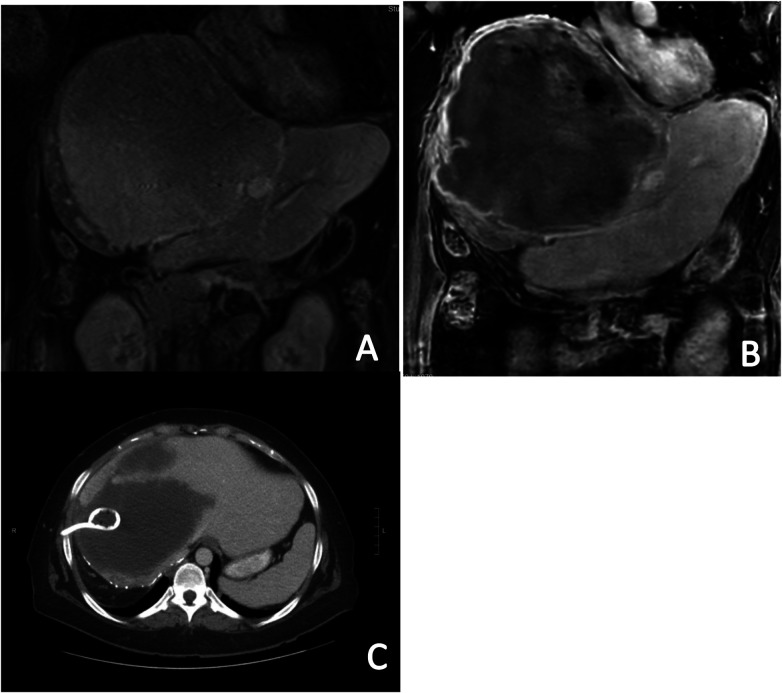
**(A)** MRI liver with contrast: a coronal view demonstrates the principal hepatic adenoma measuring up to 18 cm and causing a mass effect on the right ventricle. **(B)** An MRI liver with gadolinium: a coronal view of the main adenoma at 4 weeks after embolization. **(C)** An axial view of the immediate preoperative CT scan demonstrating a pigtail catheter in the right lobe fluid collection.

Because of the patient’s severe abdominal pain requiring narcotics and her cardiac compromise from the adenoma, the mass was embolized using embozene particles via the branches of the right and middle hepatic arteries. Immediately after the procedure, the pain transiently worsened and she developed ischemic hepatitis with a peak ALT of 3,054 U/L, AST 2,235 U/L, bilirubin 23.9 mg/dL, and INR of 2.1 that slowly alleviated over time.

Two weeks after embolization, she had an insidious onset of ascites and hemianasarca of her lower extremities with a 40-pound weight gain. She was rehospitalized and treated with bed rest and diuretics. A repeat MRI showed an interval development of two large intrahepatic fluid collections with possible hemorrhage and necrosis. The cardiac echo demonstrated normal left ventricle function but a small externally compressed right atrium with normal right ventricle (RV) filling (Figure 2). She was treated with antibiotics and sent home after being listed for LT with a lab MELD score of 22 points. She re-presented 3 weeks after embolization with fever, and a CT scan showed an enlarging fluid collection with gas measuring 17.8 × 13.4 × 17.9 cm with right atrial and vena caval impingement. A percutaneous drain was placed following the aspiration of 600 mL of bilious bloody fluid; the culture result was negative, and antibiotics were stopped.

**Figure 2:**
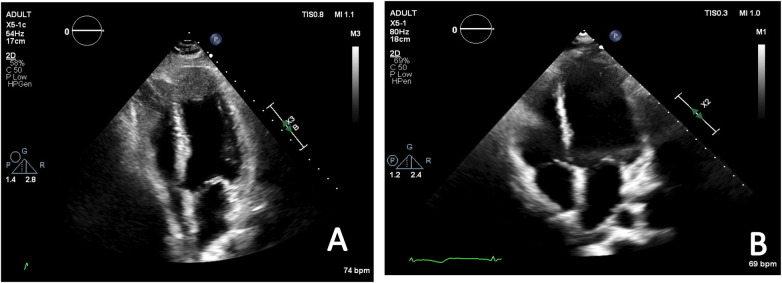
**(A)** TTE pre-LT showing external compression of the right atrium and right ventricle (Supplementary Video). **(B)** TTE post-LT showing normal filling of the right heart chambers.

The patient had weekly labs and experienced dramatic improvement in her total bilirubin to a nadir of 0.8 mg/dL. She eventually underwent a DCD liver transplant 16 weeks after initial presentation and her intraoperative transesophageal echocardiography demonstrated normal RV and right atrial filling pressures with no extrinsic compression. Because of the size of the masses, she required a bicaval transplant with standard reconstruction of all portal structures. Her postoperative course was uneventful, and she was discharged on postoperative day 7. The explanted liver showed a hepatocellular adenoma (17.3 cm) with 75% necrosis and without hepatocellular carcinoma (HCC) features (Figure 3). The background liver had features of mass effect and treatment-related changes but no fibrosis. The explant showed no findings of glycogen storage disease (Figure 1). At 15 months post-LT, she is feeling better with normal liver biochemistries on maintenance immunosuppression of tacrolimus and mycophenolate. She has had no liver-related complications and has no evidence of recurrent adenoma in her allograft.

**Figure 3:**
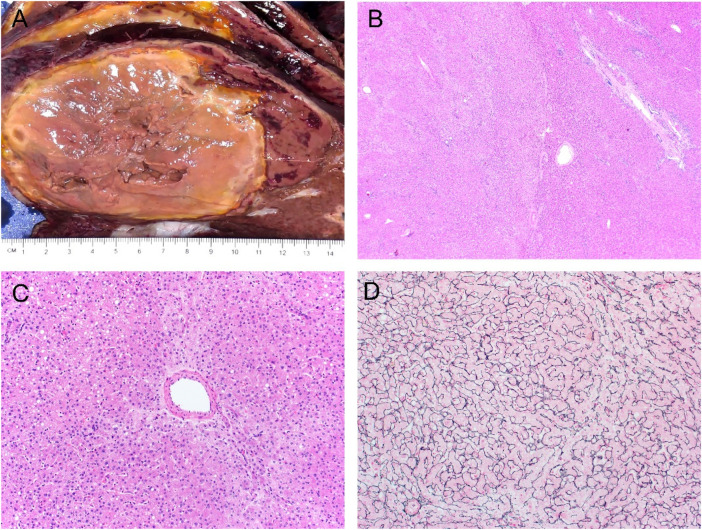
Gross and microscopic examination of the explanted liver. **(A)** An explanted liver with a large, solitary mass with extensive necrosis in the right lobe. **(B)** A well-demarcated mass with hepatocellular differentiation, unpaired arteries, and absent portal tracts adjacent to non-neoplastic, non-cirrhotic liver parenchyma (hematoxylin and eosin stain, original magnification 20×). **(C)** Mass composed of hepatocytes with no significant cytoarchitectural atypia and interspersed unpaired arteries, consistent with a hepatocellular adenoma (hematoxylin and eosin stain, original magnification 100×). **(D)** Reticulin stain shows an intact reticulin framework and no expansion of the cell plates, which, along with the morphology, glypican-3 negativity, and lack of diffuse glutamine synthetase and CD34 staining, rules out hepatocellular carcinoma (original magnification 100×).

## Discussion

Liver adenomas are highly arterialized benign liver lesions most frequently diagnosed in women aged 20–40 years (2). Risk factors for developing sporadic adenomas include the use of exogenous estrogens, obesity, polycystic ovarian syndrome, glycogen storage disease, familial adenomatosis, and congenital absence of the portal vein (Abernethy malformation). The risk of malignant transformation is low (i.e., < 1% per year) but is more common in patients with larger adenomas (> 5 cm) and in those with evidence of B-catenin expression on biopsy. The diagnosis is usually made with imaging modalities like an MRI with Eovist, demonstrating the absence of enhancement (2). Resection or arterial embolization is recommended in a minority of patients, particularly if the lesion is symptomatic or larger than 5 cm^2^. Because hepatic adenomas can recur, radiological surveillance after resection is recommended, as was done in this case (3).

The indications for LT in patients with hepatic adenomas are not well-established but frequently include mass effect, malignant transformation, or lack of other effective therapies. In our case, the patient had large recurrent adenomas that were not amenable to resection and led to symptomatic jaundice from mass effect on the common bile duct. She also had extrinsic compression of the right atrium and vena cava, leading to poor venous return to the heart, ascites, and extensive lower-extremity edema. The patient showed initial improvement after transarterial embolization of the largest adenoma. In a series (4) of 59 patients with large hepatic adenomas that were embolized, 47% had marked size reduction at 5.5 months, but seven patients (12%) had infectious complications requiring drainage similar to our patient. In that series, 25% underwent successful resection and none required a rescue LT. A systematic review (5) of 851 hepatic adenoma patients, of whom 151 underwent embolization, revealed no deaths during follow-up. However, 8% of patients experienced a complication due primarily to postembolization syndrome and/or acute kidney injury. Of note, transarterial embolization of the adenoma for bleeding or symptoms led to avoidance of surgery in 45% of the cases. The 5-year survival rate with LT for hepatic adenomatosis is excellent at 86.3% because most recipients are young and otherwise healthy (1). Of note, in only 3.6% of the 142 adenoma cases in the UNOS database was preoperative embolization reported.

In conclusion, our case demonstrates that large hepatic adenomas can cause mass effect on the heart and inferior vena cava that can be successfully managed by sequential embolization and subsequent LT. As shown in the current case, transarterial microparticle embolization of a large adenoma can lead to tumor involution and clinical improvement and serve as a bridge to definitive LT.

## Data Availability

The original contributions presented in the study are included in the article/Supplementary Material, and further inquiries can be directed to the corresponding author/s.
